# Review on the Utility of Trabecular Bone Score, a Surrogate of Bone Micro-architecture, in the Chronic Kidney Disease Spectrum and in Kidney Transplant Recipients

**DOI:** 10.3389/fendo.2018.00561

**Published:** 2018-09-24

**Authors:** Enisa Shevroja, Olivier Lamy, Didier Hans

**Affiliations:** Bone & Joint Department, Center of Bone Diseases, Lausanne University Hospital, Lausanne, Switzerland

**Keywords:** trabecular bone score, chronic kidney diseases, fracture prediction, bone microarchitecture, bone and bones

## Abstract

Chronic kidney disease (CKD) is defined as abnormalities of kidney structure or function, present for >3 months, with implications for, among others, bone health. Advanced stages of CKD have an increased risk of fragility fractures. Trabecular bone score (TBS) is a relatively new gray-level textural parameter, which provides information on bone microarchitecture and has been shown to be a good predictor of fragility fractures independently of bone density and clinical risk factors. We aimed to review the scientific literature on TBS and its utility along the CKD spectrum and in kidney transplant recipients. In total, eight articles had investigated this topic: one article in patients with reduced kidney function, two in patients on hemodialysis, and five in kidney transplant recipients. In general, all the studies had shown an association between lower values of TBS and reduced kidney function; or lower TBS values among the hemodialysis or kidney transplant patients compared to healthy controls. Moreover, TBS was shown to be a good and independent predictor of fragility fractures in patients with CKD or who underwent kidney transplantation. TBS postulates itself as a valuable marker to be used in clinical practice as an assessor of bone microarchitecture and fracture risk predictor in these specific populations. However, evidence is to some extent limited and larger follow-up case-control studies would help to further investigate the TBS utility in the management of bone health damage and increased fracture risk in patients with CKD or kidney transplant.

## Introduction

The Kidney Disease Improving Global Outcome (KDIGO) defines chronic kidney disease (CKD) as abnormalities of kidney structure or function, present for >3 months, with implications for health (1). CKD is classified based on the cause (i.e., diabetes or hypertension); glomerular filtration rate (GFR) (stage 1: GFR ≥ 90 ml/min/1.73 m^2^, stage 2: GFR = 60–89 ml/min/1.73 m^2^, stage 3a: GFR = 45–59 ml/min/1.73 m^2^, stage 3b: GFR = 30–44 ml/min/1.73 m^2^, stage 4: GFR = 15–29 ml/min/1.73 m^2^ and stage 5: GFR < 15 ml/min/1.73 m^2^); and albuminuria (normal to mildly increased: < 30 mg/g, moderately increased: 30–300 mg/g and severely increased: >300 mg/g). The presence of a higher albuminuria in each stage of GFR carries a poorer prognosis for the progression of CKD (1).

Among other health consequences, the declines in kidney function cause changes in bone mineral metabolism which weaken the skeletal system (2, 3). The CKD abnormalities that are believed to independently or collectively alter bone metabolism (remodeling or mineralization) are: secondary hyperparathyroidism, abnormalities in 1,25-dihydroxyvitamin D synthesis, phosphorus retention, chronic metabolic acidosis, and elevated sclerostin and/or fibroblast growth factor 23 levels (4–8). These abnormalities occur early in the course of CKD to become almost universal in patients with advanced CKD (9, 10). All together constitute a systemic mineral metabolism derangement known as CKD-mineral and bone disorder (CKD-MBD), a term suggested by KDIGO (3). It is well-recognized that advanced stages of CKD (stages 3a−5) place individuals at particularly high risk of fragility fractures, due to the alterations of bone strength (11–13). This increase in fracture risk is attributable to the combination of CKD-induced changes in bone and mineral metabolism, and the classic fracture risk factors observed also in the non-CKD population, such as: age, sex, previous fracture, diabetes mellitus or glucocorticoid use (14). Fragility fractures will eventually enhance the economic and social burden of CKD-MBD (14).

Therefore, the evaluation of the risk of fracture in these specific stages is very important. It is a silent agreement among the bone health community that in the earlier stages of CKD (stage 1–3), the fracture risk assessment is made as in the general population; whereas by the time a patient progresses the advanced stages (stages 4–5), bone metabolism derangements become so dominant and the fracture risk increases so much that the general population's criteria may not be relevant anymore (15, 16). Bone histomorphometry remains the gold standard to evaluate bone abnormalities in the advanced CKD-MBD (3). Nevertheless, as a costly and invasive technique, its routine clinical use is very limited. Recent studies introduced enough evidence that bone mineral density (BMD) as measured from the dual-energy X-ray absorptiometry (DXA) scans, is a good predictor of fractures in the spectrum of CKD stages 3a to stage 5 on dialysis (17–20), which allowed KDIGO for recommending BMD measurement in their 2017 update (1). However, as in the non-CKD patients, the relevance of BMD is limited due to its overestimation in the presence of scoliosis, osteoarthritis at the lumbar spine, vascular and/or joint calcifications. Moreover, it has been suggested that both bone mass and texture need to be assessed to provide an accurate assessment of fracture risk in individuals with or without CKD (14). As BMD only measures one factor, bone mass, providing no information on bone texture, which is also adversely affected in CKD, it cannot be a stand-alone risk factor for making management decisions (14).

Trabecular bone score (TBS) is a relatively new gray-level textural parameter, which provides information on bone microarchitecture (21). A homogenous microarchitecture results in a higher TBS value, whereas a more heterogenous microarchitecture results in a lower TBS value. TBS can be easily measured from the lumbar spine DXA scans, which have been previously obtained to measure BMD (21). One of the major advantages of TBS is that it is not impacted by the presence of overlaying calcifications due to degenerative changes in the bone. In the general population, lower TBS values have been associated with higher fracture risk independently of BMD and/or other fracture clinical risk factors (22–25). It is now well-established that TBS is an independent predictor of fracture risk, suggesting its use in addition to BMD in fracture risk prediction (26). As a result, TBS is now incorporated into FRAX, the most widely used fracture risk assessment tool that uses a combination of age, sex, clinical risk factors and femoral neck BMD (optional) to predict the 10-years probability of major osteoporotic fracture (hip, humerus, forearm and clinical vertebral fractures) (27). Furthermore, studies have also demonstrated the ability of TBS to predict fragility fractures in secondary osteoporosis, caused by diabetes, primary hyperparathyroidism, rheumatoid arthritis, adrenal incidentaloma, chronic kidney disease, or long-term glucocorticoid therapy (28–31).

The aim of our study was to review the current scientific literature on TBS and chronic kidney disease. We identified studies that have investigated the ability of TBS to assess bone health and to predict fracture risk along the CKD spectrum or kidney transplantation.

### Literature search

Using both free text and MESH terms (trabecular bone score, chronic kidney disease, hemodialysis and kidney transplantation), a systematic search of the literature was performed in PubMed database with no time and study type limit, until January 2018. Abstracts were selected. References of the retrieved articles were checked manually for further studies.

### TBS in patients with reduced kidney function

KDIGO defines the reduced kidney function as GFR < 60 ml/min per 1.73 m^2^ and normal kidney function as GFR ≥ 60 ml/min per 1.73 m^2^ (1). The reduced kidney function is further classified in stages 3a−4, as stated above. Naylor et al. studied the association of TBS with fracture risk in reduced kidney function and examined whether this association differs from that with normal kidney function, in the Canadian Multicenter Osteoporosis Study (32). Adults (>40 years) with reduced kidney function (*n* = 199) had a significantly lower mean TBS than those with normal kidney function (*n* = 1.227; 1.275 vs. 1.297). The 5-years probability of having a fragility fracture was higher among individuals with reduced kidney function with TBS values below the median (18.1%; 95% CI: 11.7–27.6%) than those with TBS values above the median (6.2%; 95% CI: 2.8–13.3%). Moreover, TBS showed to predict fracture independently of age, sex, FRAX score, and BMD in both groups. These results suggest that as in the general population, TBS may be a useful parameter to predict fracture risk in patients with reduced kidney function.

### TBS in patients on hemodialysis

Fractures occur more frequently in hemodialysis patients than in the general population (33). For instance, patients receiving hemodialysis therapy have a 4-fold higher incidence of hip fracture than the general population. Furthermore, hip fracture-related mortality risk is two times higher with a GFR < 45 than GFR ≥ 45 ml/min per 1.73 m^2^ (11, 34, 35). Scientific evidence has very recently supported some fracture risk predictive value of BMD in patients with end-stage renal disease (ESRD) (CKD stage 4–5) and/or on dialysis (17–20). Nevertheless, there is a lack of investigation of other bone parameters measured from DXA, such as TBS in fracture risk prediction in ESRD patients. To the best of our knowledge, two studies have evaluated TBS in this group of patients.

Yavropoulou et al. studied the possible association of TBS with other factors that reflect bone health in a cross-sectional case-control study of 50 patients with ESRD undergoing chronic hemodialysis (36). Participants were 23 women and 27 men with a mean age of 62 ± 9.6 years. The control group were 52 healthy individuals (mean age = 59 ± 9.5 years) matched for age, body mass index and gender. Patients on hemodialysis had a significantly lower TBS than controls (1.11 ± 0.16 vs. 1.30 ± 0.13, *p* < 0.001, respectively) and this difference remained significant even after adjusting for age and serum levels of parathyroid hormone, phosphate, alkaline phosphatase, and 25-OH-vitamin D3. Furthermore, TBS was significantly lower among patients on dialysis without osteoporosis than controls without osteoporosis, and this was independent of BMD or other covariates (1.15 ± 0.181 vs. 1.32 ± 0.123, *p* = 0.001, respectively).

In the second study, Brunerova et al. aimed to contribute further to the study of the utility of TBS in the ESRD patients on dialysis (37). They assessed TBS in 59 patients (mean age = 67.6 ± 13.1 years) on dialysis concluding that half of them had severely impaired bone microarchitecture as assessed by TBS. They additionally saw that there was a good correlation between TBS and quantitative computed tomography (QCT), which reflected the histomorphometric parameters. Thus, TBS seems to be an effective and non-invasive indirect marker of bone microarchitecture. Their data were too limited to further investigate the utility of TBS in fracture risk prediction.

### TBS in kidney transplant recipients

Kidney transplantation has the best prognosis for patients with severe end-stage renal failure (38). Improving the long-term quality of life for these patients is an important matter. There are contradictory results from studies that have investigated the association of fracture risk with renal transplant. Risk factors, such as duration of prior renal failure or dialysis, diabetes, corticosteroids use have been associated, but not robustly, with the increase in fracture risk after renal transplantation. Nevertheless, the field lacks a well-established way to predict fracture risk in these patients. The majority of studies on TBS and CKD, actually are on the kidney transplant patients, as the most vulnerable/exposed category of CKD patients to bone damage.

Naylor et al. investigated TBS in 327 adults kidney transplant recipients (mean age = 45.3 ± 12.4 years) who received their transplant in Manitoba, Canada; and compared it with 981 matched healthy individuals (mean age = 45.4 ± 12.3 years) (39). Kidney transplant recipients had a significantly lower TBS compared to controls (1.365 ± 0.129 vs. 1.406 ± 0.125, *p* < 0.001) and a higher fracture probability as assessed by FRAX. During the mean follow-up period of 6.6 years, 31 (9%) kidney transplant recipients sustained one or more incident fragility fractures. The recipients who sustained a fracture had a significantly lower TBS than those who did not (1.301 ± 0.144 vs. 1.372 ± 0.125, *p* = 0.003). TBS was able to discriminate between recipients with and without a fracture (area under the curve 0.64; 95%CI: 0.53–0.74, *p* = 0.012). Furthermore, the kidney transplant recipients with a lower TBS were less likely to remain fracture-free (*p* = 0.017). Finally, lower TBS was associated with fracture independent of FRAX (adjusted hazard ratio per standard deviation decrease 1.55; 95%CI: 1.06–2.27).

Peres-Saez et al. performed a cross-sectional study to analyze bone health outcomes in 53 ESRD patients (mean age = 55.8 ± 12.1 years) at the time of undergoing kidney transplant surgery (40). The control group was 77 healthy individuals (mean age = 50.2 ± 16 years). TBS was measured in a mean time of 8 days after the kidney transplant surgery. TBS was higher in the control group than in cases (1.20, interquantiles range IQR: 1.11–1.3 vs. 1.31, IQR: 1.19–1.43, *p* < 0.001). The duration of dialysis before the transplant showed to have no effect on TBS post-transplant.

Moreover, Perez-Saez et al. investigated TBS also in long-term (mean follow-up time = 10 years) kidney transplant patients in the same population with the previous study (41). The 40 cases (mean age = 63.8 ± 11.1 years) were matched with 77 healthy controls (mean age = 50.2 ± 16 years). TBS was again lower among the cases than controls, but did not reach a statistical significance (1.21 ± 0.14 vs. 1.30 ± 0.15, *p* = 0.072). Interestingly, no differences in TBS or BMD between patients on glucocorticoids and those glucocorticoids-free were found; neither a correlation between TBS or BMD with the cumulative glucocorticoid dose.

Luckman et al. followed 47 kidney transplant recipients (mean age = 50.5 ± 13.7 years) for 12 months after their kidney transplant (42). All patients underwent DXA scans at baseline, 3, 6, and 12 months after the transplant surgery. At baseline and at 12-months visit, 72 and 76%, respectively, of the patients had normal BMD—which was in contrast to TBS, as only 47 and 50%, respectively, of the patients had TBS values that corresponded to a low fracture risk (TBS ≥ 1.37). Nevertheless, 42% of the patients having normal BMD values, were classified to the high fracture risk group by their TBS values. TBS correlated to the high-resolution peripheral quantitative computed-tomography (HR-pQCT) measures of trabecular bone: trabecular thickness, density, stiffness and failure load. Furthermore, despite having normal lumbar spine BMD values, patients classified as high risk by TBS had abnormal cortical and trabecular microarchitecture and lower bone strength as assessed from the computational measures. In general, older age and a history of pre-transplantation dialysis were associated with a lower TBS at baseline; whereas male sex, pre-transplant diabetes and elevated levels of bone resorption marker (carboxy-terminal collagen crosslinks) were risk factors for declines in TBS during the follow-up period.

Most recently, Aleksova et al. investigated TBS in 146 patients (mean age = 48 ± 13 years) with progressed CKD (stage 5 on dialysis) who had undergone a kidney (*n* = 114 patients) or simultaneous pancreas kidney transplantation (*n* = 33 patients) (43). The mean TBS value was 1.345 ± 0.125 and TBS ≥ 1.31, which indicates a lower risk of microarchitecture damage, was present in 65% of the study population, whereas 15% of them had a TBS < 1.23 which indicates a high risk for microarchitecture damage. TBS did not significantly differ with sex, age or prior dialysis duration. Patients with type 1 diabetes undergoing simultaneous pancreas kidney transplantation had lower TBS values than those undergoing only kidney transplantation. Furthermore, prevalent non-vertebral fractures were significantly associated with lower TBS values (*p* = 0.023). Low TBS values (≤1.31) were a good predictor of non-vertebral fractures, but not of combined vertebral and non-vertebral fractures. Even after adjusting for the relevant covariates, TBS remained significantly associated with prevalent non-vertebral fractures independently of femoral neck BMD and FRAX (odds ratio per standard deviation decline in TBS = 1.54; 95%CI: 1.03–2.31; *p* = 0.036).

## Discussion

To the best of our knowledge, this is the first literature review of the utility of TBS in bone health assessment and fracture risk prediction in patients with chronic kidney disease undergoing dialysis or kidney transplantation. In general, these studies are in line with each other in associating lower TBS values with an impaired kidney function along the CKD spectrum and among the kidney transplant recipients; and concluding that TBS can predict fracture risk independently of bone density in this population.

The lack of a well-established strategy to assess bone health and fracture risk in patients with end-stage renal disease is an important matter of bone community. The presence of bone health deterioration in these patients could have possible therapeutic implications which could decrease the risk of having a fragility fracture and furthermore, improve their prognosis. The current gold standard method to assess bone deterioration, bone biopsy, is an invasive technique with a limited availability (3). Thus, research has been focused on the investigation of non-invasive measurements of bone strength that would predict fractures in this population. Recent studies concluded that the utility of BMD, one of the bone parameters used in the general population to predict fragility fractures and assess bone health, is valuable in the management of ESRD patients with MBD (17–20). As in the general population, BMD measurement alone cannot always estimate the severity of bone disease (14). Hence, the investigation of the utility of TBS, as another non-invasive bone parameter that have shown to be independent of BMD, in this population, has gained special attention.

In patients with ESRD on hemodialysis, TBS suggests that a significant deterioration of bone quality might be present, independent of bone mass and other biochemical changes in ESRD, postulating itself as a valuable marker in assessing bone quality in these patients (36, 37). Nevertheless, the small sample sizes of the current studies on this topic, could not allow the full investigation of TBS predictive value in the risk of fragility fractures.

The majority of studies on TBS and CKD are on kidney transplant recipients, as an exposed group to ESRD and other causes of osteoporosis, such as glucocorticoids use. They have robustly shown that patients who had undergone a kidney transplant had a lower TBS than matched controls, suggesting of a remained bone microarchitecture alteration after the kidney transplant (39, 40, 42, 43). Potential mechanisms that might explain this bone loss include the damage caused from the pre-transplant ESRD; the exposure to agents, such as glucocorticoids after the transplant, which has its major effect on bone health, affecting its microarchitecture as well; and post-operative immobility followed by increased physical activity due to improved health status (3, 44–48). Nevertheless, the only study with a long-term follow-up period of the patients, 10 years after their kidney transplant surgery, demonstrated an almost complete recovery of bone quality as measured from TBS (41). Interestingly and contrary to what was expected, this study also showed no differences in bone assessments among patients who were on glucocorticoids and those who were not—suggesting that further investigation of the long-term effect of glucocorticoids on bone in this population is needed. Furthermore, TBS was shown to be a good fracture risk predictor in this patients' category.

The wide range of TBS values in patients with reduced kidney function (32), on hemodialysis (36, 37) and kidney transplant recipients (38–43) does not allow us to speculate on an association between the deterioration of renal function and decline in TBS. Nevertheless, the results of the studies reported in this review cannot be compared due to the differences in characteristics, such as age or body mass index, and the DXA devices used in the studied populations. The exploration of this relationship and the suggestive TBS thresholds to be used in clinical practice for this patients' category in larger follow-up case-control studies would help to further elaborate on this matter.

In overall, current evidence suggests that the management of bone health alterations and fracture risk assessment among patients with CKD and those after kidney transplantation, might be similar to that of the non-CKD population. The studies robustly show a damage of bone microarchitecture as measured by TBS (and consistent with findings from other technics, such as HR-pQCT), and confirm the ability of TBS to predict the risk of fragility fractures in this population. Based on these insights, TBS assessment is reasonable in the fracture risk prediction in this population. Additional investigation on the TBS thresholds in this population will eventually contribute to the improvement of their clinical management.

## Author contributions

All authors listed have made a substantial, direct and intellectual contribution to the work, and approved it for publication.

### Conflict of interest statement

DH is co-owner of the TBS patient and has corresponding ownership and position at medimaps group. The remaining authors declare that the research was conducted in the absence of any commercial or financial relationships that could be construed as a potential conflict of interest.
